# Three Ways of Combining Genotyping and Resequencing in Case-Control Association Studies

**DOI:** 10.1371/journal.pone.0014318

**Published:** 2010-12-20

**Authors:** Jeffrey A. Longmate, Garrett P. Larson, Theodore G. Krontiris, Steve S. Sommer

**Affiliations:** 1 Division of Biostatistics, City of Hope, Duarte, California, United States of America; 2 Department of Molecular Medicine, City of Hope, Duarte, California, United States of America; 3 Medomics LLC, Azusa, California, United States of America; Washington University, United States of America

## Abstract

We describe three statistical results that we have found to be useful in case-control genetic association testing. All three involve combining the discovery of novel genetic variants, usually by sequencing, with genotyping methods that recognize previously discovered variants. We first consider expanding the list of known variants by concentrating variant-discovery in cases. Although the naive inclusion of cases-only sequencing data would create a bias, we show that some sequencing data may be retained, even if controls are not sequenced. Furthermore, for alleles of intermediate frequency, cases-only sequencing with bias-correction entails little if any loss of power, compared to dividing the same sequencing effort among cases and controls. Secondly, we investigate more strongly focused variant discovery to obtain a greater enrichment for disease-related variants. We show how case status, family history, and marker sharing enrich the discovery set by increments that are multiplicative with penetrance, enabling the preferential discovery of high-penetrance variants. A third result applies when sequencing is the primary means of counting alleles in both cases and controls, but a supplementary pooled genotyping sample is used to identify the variants that are very rare. We show that this raises no validity issues, and we evaluate a less expensive and more adaptive approach to judging rarity, based on group-specific variants. We demonstrate the important and unusual caveat that this method requires equal sample sizes for validity. These three results can be used to more efficiently detect the association of rare genetic variants with disease.

## Introduction

We address some statistical issues raised by the discovery of new genetic variants in the context of case-control association studies. We focus our attention on individual genes, partly for simplicity, and partly because the selection of cases for sequencing can be taken somewhat further in the candidate gene setting. Genome-wide association (GWA) studies are generally based on established sets of single-nucleotide polymorphisms (SNPs). Because the coverage of rare alleles by combinations of known SNPs is limited [1], an investigator may wish to identify potentially causal mutations near a GWA hit, or take a closer look at a candiate gene. There are cost-limitations on how much of the genome can be covered [2]. Even with genome-wide resequencing of expressed genes, an investigator may need a locus-focused effort to discover variation in regulatory regions, or there may me a need to probe for the newly-discovered variants in a larger set of individuals. Any of these situations is likely to raise the issues we addess.

Another general development motivating this work is the increasing interest in rare variants [3]. Pritchard [4] for example, argues that much of the genetic variance underlying disease may be due to high mutation rates into the high-risk class. While the total frequency of susceptibility mutations may be high in this situation, there will be extensive allelic heterogenity. It is also reasonable to expect that high penetrance alleles will be individually rare due to selection pressure. Dickson *et al.* [5] note that rare variants can create synthetic associations that may account for GWA results. It is an even greater problem when synthetic association is not present. High-penetrance rare alleles may be invisible to GWA studies if they are spread too evenly across SNP-tagged haplotypes. In such cases, sequencing, or an equivalent variant-discovery method, may be the only way to identify a disease association.

Throughout the paper, we consider rare alleles collectively. This is partly because their numbers may not otherwise accumulate enough to be distinguished from random events, but also because such testing associates the gene with the phenotype. The disease association of a specific SNP haplotype at a locus, presumably due to its linkage disequilbrium with undetected genetic lesions, is not necessarily more informative than the disease association of a set of rare sequences, and the former situation may resemble the latter upon sequencing of the haplotype. Collective testing permits alleles to be grouped according to an hypothesis, rather than by the accidents of linkage disequilibrium. The hypothesis might involve additional information, such as change to the protein sequence, evolutionary conservation, or initial detection in selected cases.

Even when alleles of moderate frequency are involved, collective testing has a power advantage. Slager *et al.* [6] note that allelic heterogeneity reduces the power of association tests. Longmate [7] notes that tests with multiple degrees of freedom are more powerful than tests of individual alleles, while tests of an *a priori* collection can be still more powerful, while retaining substantial robustness to misclassification. Li and Leal [8] make similar points, but add a method for combining a test of rare variants as a collective with an omnidirectional test of more common alleles. The advantage of such a combined test seems to apply when it is the common alleles, rather than the rare, that are most strongly associated with disease. Our interest being primarily in the reverse situation, we focus here on the collective testing of rare variants, deliberately excluding common alleles.

Our results address three questions. First, we consider expanding the catalogue of known sequence variation prior to genotyping a much larger number of cases and controls. This assumes that the genotyping will probe for all of the discovered variants, and not just a standard collection of SNPs. We show that sequencing can focus initially on cases, to obtain a sample of the allelic heterogeneity associated with disease, and that sequencing of controls is not always necessary.

Second, we ask to what extent can we enhance our discovery of trait-related variants by focusing resequencing efforts more strongly within cases. Even low-resolution genotyping can guide sequencing for this purpose by identifying a subset of cases who share markers with an affected relative. A general pattern we find is that the enrichment due to selection increases multiplicatively with increasing penetrance.

Our third question arises when sequencing is used to both discover and count variants in cases and controls, and we wish to focus our comparison on rare alleles. We show that genotyping in supplementary pooled samples, to establish rarity, raises no validity issues, and that the use of group-specific variant detection, which avoids the need for additional genotyping, requires equal sample sizes for validity.

## Results

Following Altshuler *et al.* [9], we distinguish three classes of allele frequencies. Common alleles, with frequencies above 5 percent, are well covered by the current HapMap. We will refer to variants with frequencies between 0.5% and 5% as uncommon. These are common enough to catalogue, but are not well covered at present. Rare mutations will be difficult to catalogue comprehensively [10].

### Sequencing cases for variant discovery

Resequencing a subset of individuals can be used to expand the catalogue of variants that less expensive genotyping methods will recognize in the remaining individuals. The use of diseased as opposed to neutral discovery panels can enrich the catalogue for disease-associated variants in the population being investigated [10], [11].

When the detection of variants is concentrated among cases, it is not valid to compare the resulting counts to those obtained by probing only for those same alleles among controls. As an extreme example, imagine a locus prone to mutation, with many unique variants. Sequencing cases would find many variants not found in controls, but sequencing controls would likely find as many variants not found in cases, and neither disparity would have anything to do with the disease.

An allele that is uncommon but not rare might be encountered several times among the sequenced cases, particularly if the cases chosen for sequencing are heavily selected. One would not want to simply discard all sequenced cases when counting alleles for an association test. Li and Leal [10] address the size of the bias introduced when variant discovery is limited to cases. Here we use a standard result in size-biased sampling to show that an unbiased test may be obtained by discarding only the first example of each allele encountered during allele discovery. Subsequent encounters may be retained.

Consider a test in which individuals are simply scored as to whether or not they exhibit a specific variant, and that the variant came to our attention through sequencing of cases, but not controls. Let 
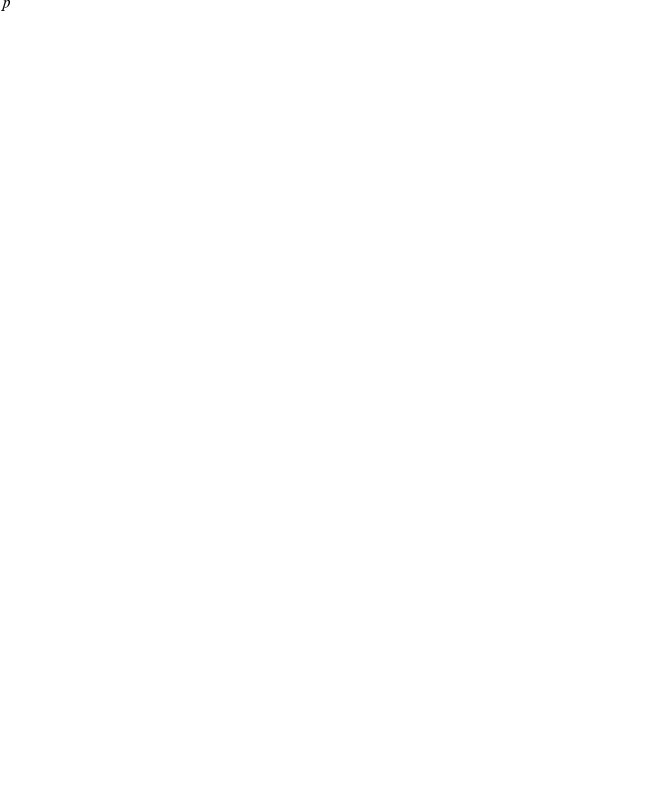
 be the probability that a sampled individual possesses the variant. Let 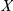 be the (possibly unobserved) number of individuals possessing the variant among the 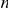 cases in the sample, and denote the binomial distribution of 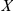 as 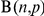. We draw a sub-sample of 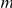 cases to be screened for allelic variants, and let 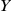 be the number of individuals with the variant in this sub-sample. If 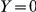, the allele is not detected, and we will not observe 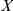. If 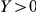, the allele will be detected and 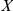 will be observed. If the allele is uncommon, and if 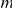 is small compared to 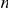, the probability of 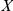 coming to our attention is approximately proportional to 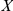, and we have a size-biased sample. The size-biased observation, 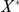 is distributed as 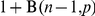 [12], hence 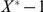 is distributed as 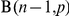. Removing the first detected case from both numerator and denominator in the observed fraction of cases yields an unbiased estimator of 
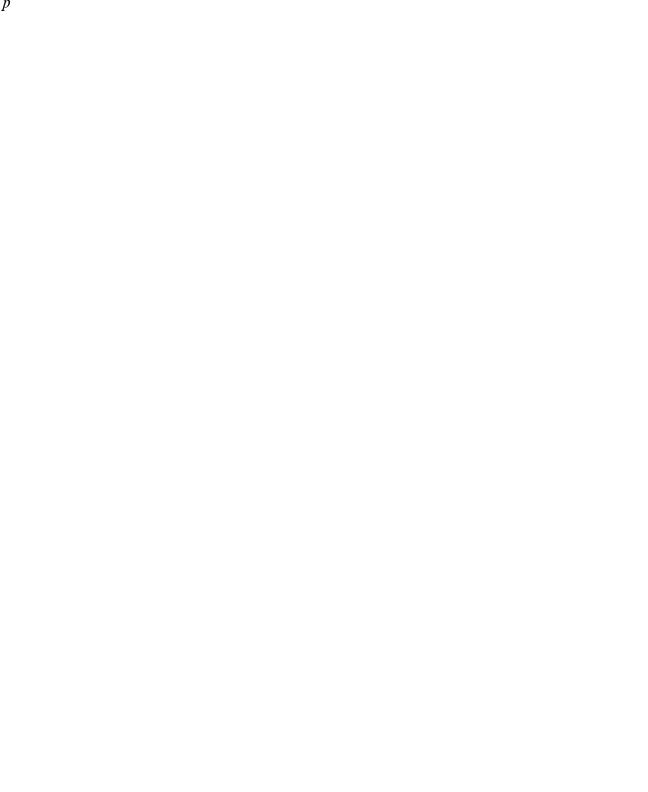
, which may be compared to the fraction of controls exhibiting the same allele, even though controls are not subjected to allele discovery.

The size-biased situation is a limiting case, applicable to rare alleles when the screened fraction is small. As we consider more common alleles, the bias due to selective screening becomes less that size-bias, and the practice of leaving out the first detection becomes conservative. If the allele is so common that we would very likely detect it in any sample of cases or controls, the fact that we have not screened controls loses its relevance.

If the number screened for variants, 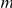, is a substantial fraction of the total number of cases, 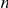, then the probability of observing the allele approaches the probability that 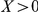, rather than being proportional to 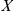. The simple omission of the first detected allele can be too conservative in this situation. If one has the ability to screen all cases for novel alleles, it is probably best to apply a similar allele detection effort to controls as well.

#### Example: ATM exon 24

As a very simple example of sequencing in selected cases we reconsider previously published data on an uncommon variant in the ATM gene [13]. The cases consist of 66 pairs of sisters with breast cancer. Controls are 126 cancer-free individuals with similar collective grandparental ethnicity to that of the cases. Allele detection was concentrated in a subset of 7 sib-pairs that shared a rare allele at the *HRAS* minisatellite locus and also shared 1 or 2 intronic microsatellite markers (*NS22*) at the *ATM* locus. The rationale was that rare alleles at *HRAS*, which are associated with a two-fold increase in the risk of breast cancer [14] as well as with an increased propensity for double-strand breaks, may interact with high-risk alleles at *ATM*, which participates in DNA double-strand break repair [15]. Exon 24 sequence was obtained for the probands of the 7 selected pairs, and two exhibited the same missense variant altering an evolutionarily-conserved *ATM* residue (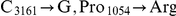).

Having seen the same variant twice in a small number of selected cases, sequence-specific genotyping was used on probands from the remaining sib pairs, as well as controls. In particular, the C-to-G polymorphism resulted in the loss of an *Alw I* restriction site, so *Alw I* digestion and gel electrophoresis were used to screen the remaining samples, identifying the variant in 7 additional cases and 4 controls, as shown in Table 1. The original report omitted all of the sequencing data from the primary calculation of statistical significance. The results above, however, show that we only need to omit the first observation of the G-to-C variant. The resulting Fisher's exact test (one-sided 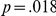) may be regarded as significant in the context of hypothesis-driven research.

**Table 1:** ATM exon 24 allele counts from 66 independent breast cancer cases and 126 unrelated controls.

		Exon 24 Alleles		Individuals	
Disease Status	Genotyping Method	C	G	variant/n	Percent
Case	Sequencing	12	2	2/7 (1/6)	29 (17)
Case	*Alw I* digestion	111	7	7/59	12
Control	*Alw I* digestion	248	4	4/126	3

The table summarizes data from Larson et al. [13]. The seven cases with sequencing shared an intronic marker at *ATM* as well as a rare *HRAS* allele with their affected sibling. Omitting the first occurrence of the variant among sequenced cases (in parentheses) permits comparing a pooled detection rate of 8/65 in cases to 4/126 in controls (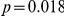 by Fisher's exact test, one-sided).

The exon 24 example only involves one variant, which is uncommon, but still frequent enough to show up repeatedly. Leaving out the first detection is obviously not an option for private mutations that may only appear once. To help understand the kinds of situations where this bias correction may be helpful, we simulated its performance in comparison to a balanced sequencing effort. Table 2 gives the simulation results for tests of the collective association of rare alleles with disease. The naive strategy means concentrating allele detection in cases only, while counting all of the alleles from the discovery set together with non-discovery genotyping. The corrected strategy refers to the omission of initial detections in the discovery set, while including repeat encounters. The balanced strategy refers to dividing the same sequencing effort equally between cases and controls. The complete strategy refers to applying allele detection, *e.g.* sequencing, to all cases and controls — which is an order of magnitude more expensive. The various scenarios represent different numbers of rare alleles, with various collective frequencies. A dominant inheritance model is assumed, and we neglect the probability of an individual exhibiting two rare variants.

**Table 2:** Test size and power using detection in subsets.

Scenario				Power (nominal 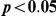 )				Detected (mid 50%)	
RR	Rare	Freq	Seq	Naive	Corrected	Balanced	Complete	Cases only	Balanced
1	20	.2	50	.08	.05	.04	.04	(7, 9)	(7, 9)
1	40	.2	50	.15	.04	.03	.04	(7, 10)	(7, 10)
1	40	.1	50	.12	.03	.03	.04	(3, 6)	(3, 6)
1	40	.05	100	.15	.01	.02	.03	(3, 6)	(3, 6)
2.5	40	.2	50	NA	.92	.89	1.00	(14, 17)	(11, 14)
2.5	40	.1	50	NA	.60	.58	1.00	(8, 11)	(6, 9)
2.5	40	.1	100	NA	.80	.85	1.00	(15, 19)	(11, 15)
5	100	.05	100	NA	.83	.89	1.00	(16, 21)	(10, 14)
5	100	.05	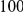 1	NA	.07	.63	.92	(16, 21)	(10, 14)

1 Number cases and controls reduced to 100, so sequencing exhausts cases.

For each line, except the last, 500 cases and 500 controls are generated in 5,000 simulated samples to estimate test size or power for a nominal 0.05-level test comparing the collective frequency of rare alleles. In each scenario, the baseline disease rate is 1%, so relative risk (RR) of 2.5 implies a penetrance of 2.5%. **Rare** is the number of unknown rare alleles in the population, all assumed to have the same frequency and penetrance. **Freq** is the total frequency of all rare alleles (*e.g.* 20 rare alleles with a combined frequency of 0.2 imply a frequency of 0.01 each). We make the simplifying assumption that rare alleles are mutually exclusive. **Seq** is the total number sequenced, either concentrated in cases or equally divided (balanced) among cases and controls. All four p-value columns are from Fisher's exact text. The first three count the number of cases and controls with any of the rare alleles detected among the indiduals that are sequenced. In the **Naive** and **Corrected** columns, all sequences are from controls, but the number of detected distinct rare alleles is subtracted from the case count in the ‘Corrected’ column. **Balanced** indicates that the individuals sequenced for allele detection were equally divided between cases and controls. **Complete** denotes the test based on sequencing all cases and all controls — a much larger sequencing effort. The parenthetic numbers indicate 25th and 75th percentiles of the number of rare alleles detected in the cases-only and balanced detection strategies.

The first four lines of Table 2 represent null hypothesis scenarios, with a risk ratio of 1. The naive test has a seriously inflated type I error rate, so it is not evaluated further. The other tests are close to or below the nominal rate of 0.05. These exact tests will be somewhat conservative due to discreteness.

In the scenarios with risk ratio of 2.5, assigning the sequencing effort to cases, or balancing it between cases and controls both lead to approximately the same power. Much higher power can of course be had by sequencing all cases and all controls, but at much greater expense. In the scenarios with higher allelic heterogeneity but fewer individuals exhibiting any rare alleles, a greater sequencing effort is necessary to maintain reasonable power.

The last line considers a smaller sample of 100 cases and 100 controls, so sequencing 100 cases exhausts the supply of cases. The corrected test loses its power in this situation. The strategy of sequencing only cases depends upon the availability of additional genotyped cases exhibiting the same alleles that are detected by sequencing. The balanced sequencing strategy maintains power in the last scenario listed. Complete sequencing has higher power, but involves twice as much sequencing effort.

### More selective sequencing

We have shown that it is feasible to focus sequencing efforts on cases, at least at the outset. We now consider how selective sequencing enhances the detection of disease-related variants, and how incorporation of family history and marker sharing can further enrich the detection sample.

For a given number of cases and controls, an association test based on genotyping will have better power if the cases have affected siblings [16]–[18]. The requirement of family history enriches the sample of cases for those with genetic risk factors, as opposed to purely sporadic cases. There is a second benefit, in that the enrichment makes it easier to discover rare disease-related alleles in the first place, so that genotyping efforts that depend on pre-identified variants will capture more of the distinction between cases and controls. Capitalizing on this aspect of family history does not require that the entire sample of cases have affected relatives. Only a subset with a family history need be identified to permit limited sequencing resources to be focused on the cases most likely to exhibit disease-related variants. In view of the difficulty of discovering uncommon alleles, and the large effect of allele misclassification on power [7], this second benefit may often be important. So we consider, in this section, the effect of various kinds of case selection directly on the probability of detecting uncommon or rare alleles.

#### Selecting Cases

If we resequence affected individuals, the probability of finding a rare allele is enhanced by a factor approximately proportional to its relative risk. More precisely, if 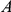 denotes the event that an individual has one or more copies of a specific rare allele, with 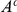 denoting the complementary event, and if 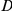 denotes the event that the individual has the disease, then the probability of observing the rare allele in a given affected individual is
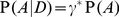
(1)


where
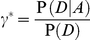



is a measure of relative risk. Another definition of relative risk is 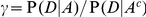, but 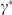 approximates 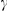 for uncommon alleles with modest attributable risk.

#### Familial Cases

Requiring the resequenced cases to have a family history will further enrich the discovery set for high-risk variants. To be definite, consider families with two siblings, with equal unconditional risk, denoted by 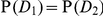, and let 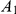 refer to the presence of a specific high-risk allele in sibling number one, whom we will arbitrarily call the proband. Then
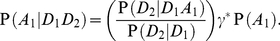
(2)


This states that the enrichment for high-risk alleles depends on the increase in risk to a sibling due to the presence of a high-risk allele in the proband. Roughly put, the sibling has about a 50 percent chance of sharing the uncommon risk allele, so the relative enrichment might approach about half again as much as that due to case status, with the two enrichment factors being multiplicative.

A little more formally, we show in the methods section that the approximation 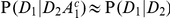, which seems reasonable if the risk attributable to a given variant is modest, together with the assumption of conditional independence of 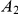 and 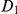 given 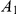 (or 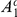), leads to
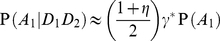
(3)


where
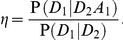



Here 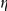 is a measure of relative risk due to the allele in the presence of an affected sibling. For an allele that confers risk rather than protection, we have 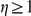. If the other sources of risk to a relative are limited, 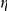 might approach 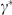, but if alleles at other loci are important, 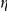 may be closer to 1, and further selection might be needed to enrich the sample for risk alleles at the locus of interest.

#### Marker-Guided Case Selection

Selecting familial cases enriches the sample for cases with a genetic etiology relative to sporadic cases, but if there is genetic heterogeneity, the enrichment may be spread across several genes. Further enrichment for high-risk variants at a specific locus is possible by focusing resequencing on cases that share one or two copies of a marker tightly linked to that locus. It is only necessary that the markers identify whether the relevant genomic region is shared identical by descent (IBD). The markers do not need to be in linkage disequilibrium with any risk alleles.

Because multiple selection criteria bring many details into play, we explore their effect via numerical calculations based on explicit scenarios. This is an extension of a previously described method [7] (see methods). As above, we assume each proband has exactly 1 sibling at risk, with the joint probability of disease denoted 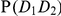. A scenario consists of a model for the probability of disease as a function of genotype, along with allele frequencies for each gene in the model. We include a background rate of disease which may represent sporadic cases, or the effect of genetic background. We initially focus on a single locus, then briefly investigate the impact of a second locus.

Table 3 gives the probability that an uncommon risk allele is present in various strata, representing different levels of selection. We consider two alleles and suppose there is a one percent disease rate in individuals homozygous for the major allele, and a higher rate in individuals with one or two copies of the minor allele, the ratio given by 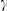. We consider a minor allele frequency of 0.005, so the minor allele is present in about one percent of the population under study. The ratios of probabilities with different levels of selection depend strongly on the relative risk, 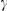. These are, however, quite insensitive to both the absolute minor allele frequency and the baseline probability of disease (data not shown). The baseline probability of 0.01 was chosen to permit easy interpretation.

**Table 3:** Detection Probabilities for High-Risk Variants.

Selection	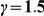	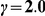	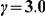	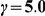
**Single gene model**				
All Individuals	0.010	0.010	0.010	0.010
Cases	0.015	0.020	0.029	0.048
w/affected sibs	0.019	0.029	0.057	0.130
sharing 1 or 2 IBD	0.020	0.032	0.066	0.155
sharing 2 IBD	0.022	0.039	0.083	0.201
**With a second, nuisance locus**				
All Individuals	0.010	0.010	0.010	0.010
Cases	0.014	0.019	0.028	0.045
w/affected sibs	0.017	0.026	0.049	0.109
sharing 1 or 2 IBD	0.018	0.029	0.056	0.130
sharing 2 IBD	0.020	0.034	0.070	0.169

In each scenario there is a sporadic disease rate of 1% and the high-risk allele of interest elevates the disease risk by a factor of 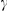, which varies across columns. The rows represent increasingly restrictive sampling rules, and the probability that the high risk allele is present (one or two copies) in a sampled proband is tabulated. In the upper half of the table, risk depends only on one locus. In the lower half, there is also a nuisance locus, with a 5 percent allele frequency, *i.e.* 10 times as common as the allele of interest, and additive with its effect.

From Table 3 we see that an allele present in one percent of a population will, if it confers a doubled risk, be present in about two percent of cases, about three percent of cases with affected siblings, and about four percent of affected sibling pairs sharing two alleles IBD at the locus. The median number of resequenced samples needed to detect the minor allele would drop from 69 in unselected cases, to 38 in cases, to 18 with marker sharing. The factor by which increasingly selective resequencing enhances the detection of disease-associated variants increases dramatically with the relative risk. If rare alleles do tend to have high penetrance, then selective resequencing seems likely to pay off well.

We examined the robustness of these results by modeling the effect of a second locus with a somewhat more common high-risk allele, also with a dominant effect, that adds 1 percent risk (a two-fold increase over baseline). The second locus can also represent two classes of genotype, as might occur if there are several dominant alleles with similar penetrance. The results are shown in the lower half of Table 3. The ability of case selection to enhance the detection of the rare high-risk allele at the locus of interest was somewhat attenuated under this additive model.

If the variant (or class) at the nuisance locus is known, then selection for the absence of this alternative etiology would substantially restore the enrichment. A similar idea seems to have guided an investigation of the CHEK2 gene in breast cancer that was carried out using non-carriers of BRCA1 or BRCA2 mutations [19]. Further calculations (not shown) indicate that exclusion of cases with a likely alternative etiology can enhance the detection of rare alleles under an additive model. Under an interaction model, however, detection of the rare variant can be enhanced by including, rather than excluding, the cases with the high-risk variant at the second locus. This behavior is consistent with the previous discussion of the limits of the parameter 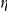 in equation (3). The correct use of information from a second locus of established relevance depends on its relationship to the locus under investigation. If genetic heterogeneity is more likely than strong positive interaction, then omission of cases with an explainable etiology would be the preferable strategy. In the ATM exon 24 example, described above, we selected cases sharing a rare HRAS allele and one or two ATM alleles with an affected sibling, on the hypothesis that the functions of HRAS and ATM would imply an interaction.

### Focusing on Rare Variants

Cases-only sequencing and selective sequencing are useful for uncommon variants. The study of rare variants requires resequencing of all cases and controls. We may then wish to focus exclusively on rare variants, motivated by the hypothesis that rare variants are the primary source of risk. Focusing exclusively on rare variants would then avoid diluting the effect size of a collective test, and may elucidate a class of variants of some predictive value.

One can focus on rare variants by omitting alleles that are known from a sequence database, but neutral alleles of modest frequency may not have been catalogued. We consider two approaches for limiting attention to rare alleles. One approach is to limit attention to group-specific variants, *i.e.* sequences that only appear in cases or only appear in controls, but not in both [20]. This would include both rare mutations and uncommon mutations of high penetrance. The other approach is to use genotyping in pooled samples to screen for the putative rare sequences in a very large number of individuals [21]. This approach can economically focus attention on what are essentially private mutations, without regard to penetrance.

#### Group-specific sequences

An advantage of comparing the frequency of group-specific variants is that a variant may be restricted to cases because it is rare, or because its penetrance is sufficiently high that it does not appear among the controls. The main disadvantage is that the validity of the comparison depends on equal numbers of cases and controls, as described below. If there are variants that confer protection as well as risk, both will tend to be included, but this is a fundamental problem of testing a collection of rare variants.

To demonstrate the requirement for equal sample sizes, consider a population containing a set of rare variants indexed by 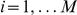. Let 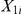 be the number of detections of variant 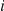 among 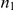 cases, and let 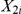 be the number of detections among 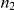 controls. The group-specific inclusion scheme amounts to simultaneously replacing 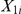 by 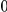 if 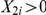, and replacing 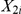 by 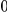 if 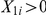. We can compare cases to controls by computing
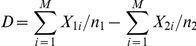



and comparing 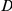 to its standard error. Under the null hypothesis, for a given 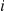, 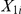 and 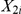 have independent binomial distributions, with common rate 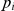 and indices 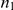 and 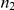. Denote these binomial probability functions by 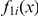 and 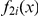. Then the weighted joint probability function, after applying the selection criterion, is 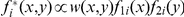 where 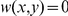 if both 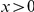 and 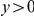, and 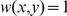 otherwise. Substituting the binomial probability for 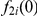 and summing over 
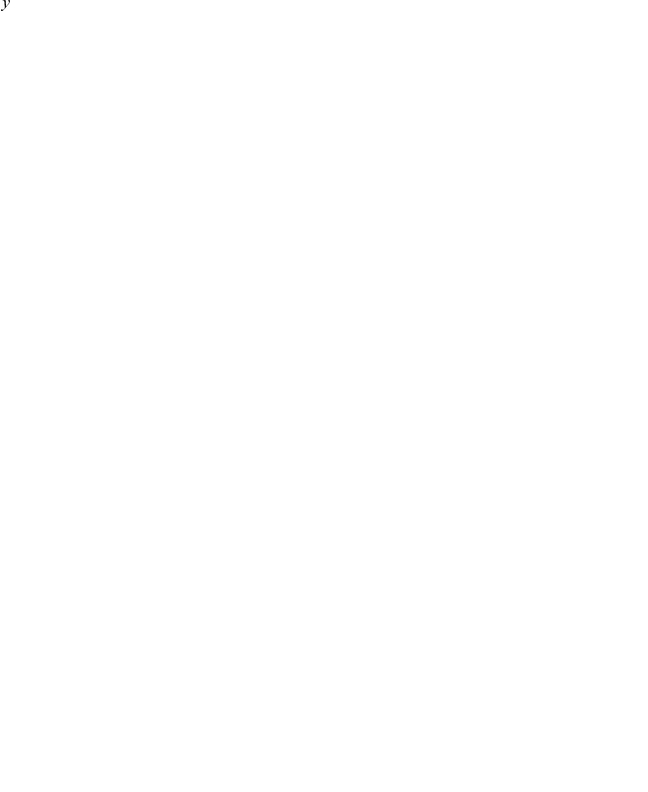
, we obtain the marginal probability function for cases,
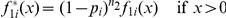



with the remaining term, 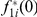, determined by subtraction from 1, and a similar expression for 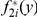 applying to controls. It is apparent at this point that the condition 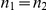 is sufficient to ensure that 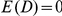 under the null hypothesis, *i.e.* our estimate of the difference in rare variant frequency is unbiased.

If 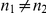, however, there can be a substantial bias. As a demonstration, we generated allele frequencies for a hypothetical locus with 100 variants ranging in frequency from approximately 0.6 to 0.00006. Because of the uncertainty about the likely distribution of rare alleles and our limited purpose, we arbitrarily took variant frequencies to be 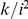 where 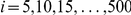, and the normalization constant, 
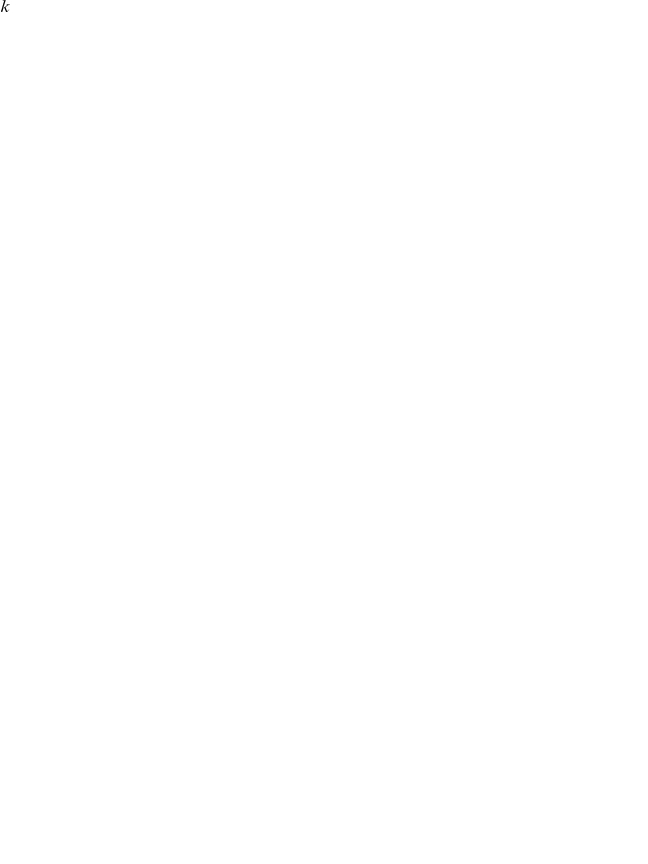
 is 15.2. This resulted in approximately 83% of the total allele frequency in 3 common alleles, 12% in 8 uncommon alleles, and 5% in 89 rare alleles, including 1.9% in the 76 with detection probability less than 0.001. If 1000 case and 1000 control sequences are sampled under the null hypothesis, many of these 100 alleles would not be detected, and the expected count of group-specific detections is 16 in cases and 16 controls, based on a Poisson approximation. If there is a marked difference in sample size, say 1200 case and 800 control sequences, we expect 22 group-specific detections among cases, but only 11 among controls, the expected rates being .0185 versus .0141, for a bias of approximately 27 percent (%L [22], calculated as 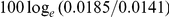). The bias arises because the larger group will exert a disproportionate suppression of the counts in the smaller group. With a more modest imbalance of 950 cases and 1050 controls, the bias is about 7%L.

Adding another 100 very rare alleles (with little change in the total frequency of common, uncommon and rare alleles) increased the expected number of group-specific dections from 16 to 19 per group, but had little effect on the bias (23%L with 1200 v. 800; 6%L with 1050 v. 950). The bias is mainly determined by the uncommon alleles that may or may not be encountered more than once.

The amount of bias depends on the specific set of allele frequencies, as well as the degree of imbalance, but this arbitrary example shows that the bias can be appreciable. The comparison of group-specific variants, while appealing in concept, seems to require rather close matching of sample sizes.

An excess of group-specific variants at the *ATM* gene has been reported in breast cancer [20]. Comprehensive *ATM* mutation screening of all coding exons and splice junctions has been carried out using the DOVAM-S [23], [24] method, which is comparable to sequencing for mutation detection. Variants affecting protein structure or expression were found in 23 of 90 women with breast cancer, and 13 of 90 women without breast cancer (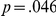, one-sided Fisher's exact test). The association of group-specific variants with breast cancer was stronger, finding 14 in 90 cases, but only 4 in 90 control samples (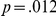). We speculate that the stronger group-specific result reflects higher average penetrance due to better exclusion of neutral variants from the collection. We also note that collective testing is predicated on a common direction of effect, which implies that one-sided tests are appropriate.

#### Pooled-sample screening

A more laborious but more direct way to restrict attention to rare alleles is to limit the events counted among cases and controls based on a third sample. Highly sensitive detection methods [25] now make it possible to follow the sequencing of cases and controls by probing for additional instances of the newly detected variants in a very large number of individuals through the use of a feasible number of pooled samples. This approach has been used to demonstrate that rare missense variants found in schizophrenia patients were not present in 10,000 control alleles [21].

We can represent this strategy by writing the joint distribution of detections for sequence 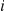 as 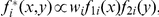 where the ascertainment weight function, 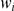 is 0 if the sequence 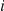 is detected in the third sample, and 1 if it is not. This is not dependent on either 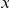 or 
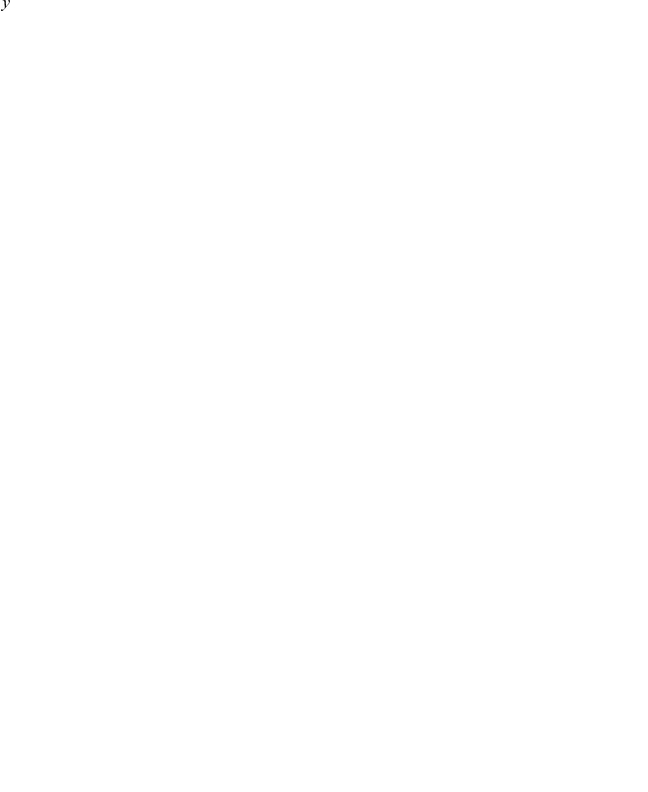
. Under the null hypothesis, 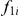 and 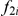 are binomial distributions with a common rate parameter, 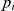, so 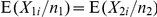 for all 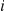, hence 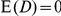 under the null hypothesis.

The selection of sequences absent from a supplementary sample eliminates some of the terms in both sums contributing to 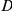, but it does not distort the distribution of any of the remaining terms, and so leaves the estimated difference unbiased for the the correct value of zero under the null hypothesis. The value of the selection depends on the alternative hypothesis, *i.e.* on the correctness of the belief that rare alleles will tend to have a larger average penetrance, and therefore a larger relative difference in detection rates. The power benefits of selecting rare alleles may depend on the details of this assumption. The point here, however, is that selection of sequences based on a third sample does not raise any validity issues.

## Discussion

In this paper, we provide three results of wide applicability rather than a prescriptive method. We focus primarily on a simple case-control setting, and a simple test of the collective effect of uncommon or rare alleles.

At present, resequencing on a substantial scale needs to be targeted to a specific locus [26]. Attempting to expand the catalogue of genetic variation at a locus might be encouraged by a belief that rare alleles will have larger effects than common alleles. The finding here lends further encouragement, in that higher penetrance is likely to translate into easier detection among cases, with the effect of penetrance being magnified as resequencing becomes more selective. Focusing sequencing efforts on the subset of cases that report a positive family history can increase the yield of disease-associated variants, a phenomenon closely related to the power advantage that has been noted when cases have affected relatives. The advantage of focusing limited sequencing resources can be obtained, however, by the opportunistic enrollment of siblings, or even by simply collecting family history data. It does not necessarily require any efforts to enhance the recruitment of familial cases, although the power advantages of such efforts still apply.

The identification of a subset of cases most likely to harbor disease-associated variants at a locus may permit the use of a more powerful test statistic that capitalizes on the hypothesized ordering of controls, cases, and select cases, with regard to the prevalence of rare variants. A test for a trend in proportions can be applied to the data in Table 1, for example, but due to the adjustment for cases-only sequencing, the result is not much different from that comparing all cases to controls. If the same results had come from sequencing, however, without need for adjustment, the p-value would be reduced to .004 (exact Cochran-Armitage test), reflecting the enrichment for rare variants in the selected group. While the use of marker-sharing for case selection is only applicable to locus-specific sequencing, the sharing of markers among affected siblings can be used as part of the statistical analysis in genome-wide sequencing studies. Madsen and Browning [27], for example, propose assigning scores to individuals, based on the number an type of mutations present. Including marker-sharing information in such scores would be a natural extension, although it would complicate the null distribution, taking the idea beyond the scope of this paper. The idea does, however, suggest how to extend the use of marker sharing from the the locus-specific sequencing setting considered here, to the setting of exome or genome sequencing.

The need to discard first encounters when combining cases-only sequence data with case and control genotyping has different implications in different situations. When many unique variants are encountered, extensive resequencing of both cases and controls is necessary. When most variants have sufficient frequency to be repeatedly detected, the simple bias correction of omitting the first encounter is competitive with a balanced resequencing effort. This, together with the expense of resequencing, makes it natural to start with cases, perhaps supplemented by family history and marker data. If multiple uncommon alleles are encountered more than once, an expansion of the study using genotyping might be indicated. If mostly rare alleles are discovered, without repeated detection, a larger resequencing effort that includes controls might be planned.

Whether the comparison of cases to controls should be restricted to rare variants, common alleles, or both, may depend on previous work, and the attitude of the investigator about the likely effects of rare or common variants. There does not seem to be much difficulty with the use of external data to select the alleles of interest, provided that the supplemental data are not used as frequency data in the comparison. Selecting rare sequence variants based on group-specific detection provides advantages of economy and adaptively relaxing the rarity requirement as penetrance increases. It is important to note, however, that this approach requires equal sample sizes for validity.

## Methods

To derive equation 3, we assume that the variant of interest is sufficiently uncommon that we may neglect bi-lineal inheritance. Then we can write
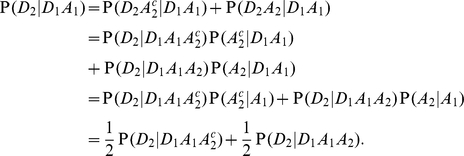



The first equality follows from the law of total probability, the second from the definition of conditional probability, the third from assuming conditional independence of 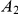 and 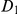 given 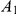, and the last from neglecting bilineal inheritance of the rare allele. The symmetry of the sibling labels means that we are also assuming that 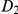 is independent of 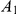 given 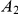 (or its compliment), so
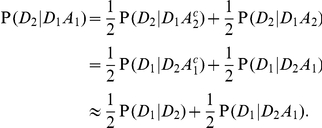



The second equality follows again from the symmetry of the siblings, and the approximate equality follows from our initial assumption that 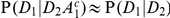. Substituting for 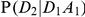 in equation 2, and observing that 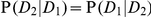, yields equation 3.

In order to consider the effects of marker-based selection of cases, we explicitly compute the joint distribution of genotypes in a pair of siblings, and their joint disease status, conditional on the number of genes shared identical by descent (IBD) at a specified locus. This is an extension of a previously published renormalization method for calculating case and control allele frequencies from prospectively specified models [7]. Briefly, Let 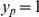 if the proband is affected, and zero otherwise. Let 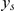 be similarly defined for the sibling. Let 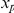 and 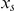 represent the proband and sibling genotypes, Let 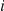 be the number of alleles shared identical by descent at a given gene, with probabilities 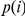. The conditional distribution of proband genotypes is given by
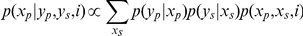



where the penetrance model specifies 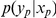, which is the same function as 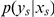, and where 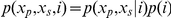 is easily calculated assuming Mendelian segregation and Hardy-Weinberg genotype proportions. (We are here abusing notation to let arguments distinguish probability functions.) By working with ibd status directly, we approximate the effect of selection based on highly informative markers.
